# Supplementary material for: Multiplex Editing of the Nucleoredoxin1 Tandem Array in Poplar: From Small Indels to Translocations and Complex Inversions
Source: CRISPR J. 2023 Aug 14;6(4):339–49. doi: 10.1089/crispr.2022.0096 (PMC10460964; doi:10.1089/crispr.2022.0096)
Supplement: Supplemental data [file Suppl_TableS1.pdf]

Table S1. Primers used in this study.

Target	Primer sequence (5' to 3')	Purpose and note
U6-gRNA	F GTTTTCCCAGTCACGACGTTGTA<br>R TGGCTTGCTATTCCCTTCTCAAGCCTACTGGTTCGCTTGA	CRISPR vector construction
gRNA-SF	F GAGAAGGGAATAGCAAGCCAGTTTTAGAGCTAGAAATAGCAAGTT<br>R CGCACAGATGCGTAAGGAGAAA	CRISPR vector construction
All PtaNRX1s	F GCAATTGTTCTAGCMTTTTGAGT<br>R AAGTTCGTGYTTCACTTTCTCTG	Multigene PCR, Figs. 1C, 2E, 3B
All PtaNRX1s	F TTGGCAATTGTTCTAGCCTTT<br>R GATGYTCCTCGGTAAATGGAA	Multigene PCR, Fig. 2C
All PtaNRX1s	F CCTACACGACGCTCTTCCGATCTGAGGTGGTSTTCRTTTCTTCTGA<br>R GTTCAGACGTGTGCTCTTCCGATCCCTCTTACTTTGAAYAMTTYCT	Amplicon sequencing Set 1
All but PtaNRX1.2	F CCTACACGACGCTCTTCCGATCTGARKTGGTBTTCATTTCTTCTGA<br>R GTTCAGACGTGTGCTCTTCCGATCCATCCACRCCATGYTC	Amplicon sequencing Set 2
PtaNRX1.2	F CCTACACGACGCTCTTCCGATCTGARKTGGTBTTCATTTCTTCTGA<br>R GTTCAGACGTGTGCTCTTCCGATCTGGCCAATTATGACAAGATTAGGG	Amplicon sequencing Set 3
All PtaNRX1s	F CCTACACGACGCTCTTCCGATCTGGGABCCCTCTTACTTYGAA<br>R GTTCAGACGTGTGCTCTTCCGATCGCAATTTGGTTGGGAAGATTGT	Amplicon sequencing Set 4
PtaNRX1.1	F GARKTGGTBTTCATTTCTTCTGA<br>R CATGCGACGTTAGCAGAG	Gene-specific PCR
PtaNRX1.2	F TGATTCGTTAAGGGCTGATGTT<br>R TGGCCAATTATGACAAGATTAGGG	Gene-specific PCR, also amplify 1.1/1.3/1.4/1.5 with one to two mismatches
PtaNRX1.3	F TGATTCGTTAAGGGCTGATGTT<br>R TCACAGCACATCGTATGGATT	Gene-specific PCR, also amplify 1.5-1.7, 1.5-1.3, 1.4-1.7, and 1.1-1.7 fusions
PtaNRX1.4	F CTTGTCTTATTATAAGTTGACGTCTT<br>R ATGCAAGGTTAGCATAAAACAAG	Gene-specific PCR
PtaNRX1.5	F TTTATGATTAAGAAAAAGCTCCCT<br>R ATCTGCCATTACAGATAGCAAG	Gene-specific PCR
PtaNRX1.6	F TTTGTTTACCCTCTTRATTTAWGGG<br>R CACATCCKATGASCATRSGCTG	Gene-specific PCR
PtaNRX1.7	F TTTGTTTACCCTCTTRATTTAWGGG<br>R TCACAGCACATCGTATGGATT	Gene-specific PCR
PtaNRX1p$_{c10.1}$	F CGGAGACTCGCAAACTTCTT<br>R GGGATCTGCCACTTCTAATAGC	Gene-specific PCR
PtaNRX1.7-1.2 & 1.6-1.2 fusions	F TTTGTTTACCCTCTTRATTTAWGGG<br>R TGGCCAATTATGACAAGATTAGGG	Fusion-specific, also amplify PtaNRX1.6 and PtaNRX1.7 with two mismatches
