# Supplementary material for: Multiplex Editing of the Nucleoredoxin1 Tandem Array in Poplar: From Small Indels to Translocations and Complex Inversions
Source: CRISPR J. 2023 Aug 14;6(4):339–49. doi: 10.1089/crispr.2022.0096 (PMC10460964; doi:10.1089/crispr.2022.0096)
Supplement: Supplemental data [file Suppl_TableS2.pdf]

Table S2. xGen lockdown probes used in NRX1-capture sequencing

Probe Name	Sequence
probe_01	ATAGCATTTGGAATCGGCAATTGTTCTAGCCTTTTGAGTAGTTCATCACTTAACAATGGCCAACGAAGACGTTTCGCACGACCTTTCATCGCTTCTTTCGTCGGAAGAGAGGGACTTTCT
probe_02	AAGAGAGGGACTTTCTCATCCGCAACAATGGTGACCAGGTACTGCTTTTAGCTTATGGATTTCTGTTTTTCTCGTAATTAAGATGGATATCATGTTTAAATTAGACTGCGAACCTTGCTG
probe_03	ACTGCGAACCTTGCTGAAGAACAAGTTTATGACCAATTTCTAGAGCTTTTATGATTAAGAAAATCCTCAATTTTTGTTTACCCTCTTAATTTAAGGGCTAATGTTTTTTCTGATTATGCT
probe_04	TTTTTCTGATTATGCTCCACGCTAATTTTAGGTTAGGGTCAGCAATTTGGTTGGGAAGATTGTGGGATTCTATTTCTCTGGTTATTGGTGCGGGCCGTGCCGTAATTTCACTCCGTTGTT
probe_05	ATTTCACTCCGTTGTTGGTAGAAGTCTATGAACAGCTATCATCCAAAGGGGACTTTGAGGTGGTCTTCATTTCTTCTGACGGACACGATGAATTCTTCAACACATACTTCTCCGAAATGC
probe_06	TGATACGGAGACCCGCCAACGTCTTAAGAAAGTGTTCGAAGTAAGAGGGATCCCTAATCTTGTCATTTTTGATACGAATGGCAAGGTTTCATGCGATGATGGAGTCAGCACTGTCATGGA
probe_07	CATGGAACATGGCGTGGATGGGTATCCGTTCAACCTTGATAGACTGATTTTCCTGAAAGAGCAAGAAGAGAAAGCTAAGAAGAATCAAACCATAAGCTCTATCTTGGTTTCCAGCTCACG
probe_08	CTCACGTGATTATGTGATTTCAAATGATGGAAAAAAGGTAGATGGTTATTCAATTAGCTAGTTTTGAAATTGAATTGATGATGCCATAATTCACATTTTTTCTATCTGTAATGGCAGATC
probe_09	CAGATCCCTTTGTTGGACCTTGAAGGGAAATTGGTTGGCTTGTATTTTTCAATCCATACGATGTGCTGTGAATTCACTCCAAAATTAGTGGAATTGTATAAGACGCTCAAGGAAAGAAGA
probe_10	AGAAGAGAGAACTTTGAAGTAGTCCTAATATCTCTAGACGACGAAGAAGAAGACTTCAAAGAGAGTTTTGAGACAATGCCTTGGTTGGCATTGCCTTATAAGGACAAGAGCTGCGAGAAG
probe_11	GAGAAGCTAGTGCGGTATTTTGAACTTAGAACCATTCCTAATCTTGTCATGATTGGCCAAGATGGGAAGACTTTGAACCCAAATGTAGCTGAACTGATCGAAGAACATGGTATTGAAGCC
probe_12	GAAGCCTACCCATTTACACCGGGAAAGCTTGACGAGCTAGCTGCAATTGAAAAGGCAAAACTGGAATCGCAGACGCTTGAGTCAGTTTTGGTTAATGGGGAAAATGATTTTGTGATTGAC
probe_13	ATTGACAAAAGTGGCTCCAAGGTAATTGTTTTTGTTCTATGCTAACCTCGCATGTTGTTACCGCATTACTTTCAATAACTTTCTTATGATCACGAATTGAATTCATTAGCAAAGGGTTTT
probe_14	GGTTTTTTTTTTTTTTATCATTCAAATGGTGTGCTCTTGATTAGGTCCCAGTGTCTGAACTAGTTGGAAAGAACATTCTTCTTTACTTCTCAGCTCAATGGTGCCCTCCATGTCGTGCCT
probe_15	GTGCCTTTTTACCCAAGCTAATTGAAGCATACCACACAATTAAAAGAAAAGACAATGCATTTGAGGTGATCTTCATCTCAAGTGACAGAGATCAATCCACCTTTGACGAGTTCTATTCAG
probe_16	ATTCAGAAATGCCTTGGTTAGCCCTTCCATTTGGTGATGAAAGGAAACAAATCCTGAGTCGGAAATTCAAAATTCAAGGCATTCCTGCAGCTGTAGCGATTGGGCCAAGTGGCCGGACCA
probe_17	GGACCATTACCAAGGAAGCTCGGATGCACCTGACAGCTTACGGGGCAGATGCTTTTCCATTTACCGAGGAACATCTAAAGCAATTGGAGGAGGAGCTTGAGGAAAAGGCAAAGGGGTGGC
probe_18	GGTGGCCAGAGAAAGTGAAACACGAACTTCATACTGAGCATGAGCTGTTACGTAGTAAACGCAAAACATATATTTGCGATGGCTGTGGGGAAACAGGAAATAGTTGGTCTTTCCATTGCA
probe_19	ATTGCAAAGAATGTGACTTTGATCTTCACCCCAAGTGTGCTTTGAAGGAAGATGAAGATACTGGAAGTGAAAAGGGAAATGAAGGATGGAACTGCGATGGAGATGTGTGCCGCCGAGCCT
probe_20	GAGCCTAAGCAGGCAAGCGCTCCTTGTATCTGGTTTTTTGGGCTTTCTGCTGTAATAAGCGTGCGATGTAAGGTGTTTGTTGCATGTGTGTGAAGATGTGGATGTCTTTCCTGTTATTGA
probe_21	TATTGATATGTAATTAAGAGGTGTGTTGCTTTAGGATTTTCATGTCCTGTATTTCGATACGAAGAATAAATAACCGGATTGGCTAGGTTTATGTGAATCCTGTGACCCTTTTGAAAC
probe_22	CTGGAAGAGGTGCTATGCATGGTTCCATTGTGCCACTCTATTTGTTTCTTTTTGGCTGGATGAAGCAGCAGCTTCACCTCTGGTGCAAATATTCCTGTAATTGTTCTGCAGATTAAAAAA
probe_23	AAAAGATGGCTGATTGTGACTAAACCTACATTATATAATGAAACCTTTCTAACTCCACCGTAAAAGAAACAAGAATCTCCGATCCAAGCAACTGGATAGTCTCTTTTTTCTCTCTCTCTG
probe_24	TGCCTTCTCACATTACCTGTTGAGGCTGGTTTCAGAATCAAGAAAGCGGCATTAATCTTAACTCAGAACTAAGCAAGAAAAACTCATCCAATAGAAATGGCAATTCTTCTGTTGCTGATG
probe_25	TGACTGGTTGACAAATGAAACAACAGCAAGTGGAAACTGGGTTCATTACAGTTATTTGAATGGCGCTAAGGGTGGAACCAAAATCACGAGCAGGCTATTGCTTGTACCAGTCGATGGGAG
probe_26	AGGCAAAGGACGGGTAGGATTAATGGCGAGGTTCTAGAAATTGGTGAAGCCAACAGCTGTACCAAGAACACTGAGATGATGGTAAGAGCTTATTTGTTATTCTGTTAATGTTTTTTTTTC
probe_27	TCTTTTAAGTGTTTTTAAATTGAAAATATTTTAAAATAATATTTTTTATTTTTTAAAATTTATTTCTCATTTCAACGTATCAAAATAAAAGTAATCTAAAACAAAATAAAAATTTATTTT
probe_28	TTAAAATATTATTAAAAAATAATTTTAAAACACTAAAAATAATGTAATCTAAAACAATTTTTTTCGAAGATTTTTTTCAATCTAAAAAACAACATGCCTAAATAATTTACTTTACTTGTG
probe_29	TGTGTGTTTAGTTTTAAAATTCAATTCAGCTAAATATGTTAACCTGAAGCTTGAACCAGGCCGTGTTTATAAAAAAATTAAAAAGGTTAAAAATGTCTCGTGTACTACAAAAGTTTTATC
probe_30	TCTTTAAACTTTGTGTAATGATACGACTAGTAAAACCTGTCAAAACTTAATTATAATTATTTAACTTTATATTTTTCATAAAAAAAAAAAGTATTACAAGTTTAAAAAATATACTTGTTA
probe_31	TACCAAAAAAAAAACTCAAATGAGTTAACTTTGTTTGCAGTGAGAGTAAGAATTTTTGTTATATATATATATATATATTCAAAGAAACTACAAAAGGTAAGGTTATGAAATAAAAAGGC
probe_32	GCAAGTTAGAAAGTGCAGAAGAAATTTTCTTGTTAGTTTTTAAATTTTATATTGTAGAATTTATGTCTACCAATTCATATTTAATAATAAAAATTAACCCGTCCCTAGATTTACTTGATA
probe_33	TATTTAATTTTAAAATTTAAAATAAATAATTAAGGTGCACGCTACATCGGTTACTTTAAAAAACATTTGAAAGGGTGTGTATCCTAATCTCCACCGTTAATCATACCGTCCATGAGTCTG
probe_34	TGATCGCACGGTTTTTATAACATCTTCAGTCTTCTGTCAGTATATTCCAATTTTGCAGATCAAATCATATCTCTCGCTTTCTCGGTAAGATTCTTCCTCTCTCTCTCCTTTACCATGCGA
probe_35	GATCTGCGTTTAGAATTATTACTTCAATCCTTTCGTTAATTCCTTAATTATGATCTAGGGTTTTGGGGTGTTAATTAAATTGAATTATTATGTTTGGGTGATCGTTGTAATTTTTGGTGG
probe_36	GGTTAGATGGATTGGCAAGGACAGAAACAAGCGGAGCTATGGATGCAGATACTGCTGTTAGTTTTCGCGGCGGCGGCTTTAGTTATAGGTTATATAATAGGATCGTTTCGAATGATGATG
probe_37	TGCTAATTTACGCCGGTGGAGTGGTTTTCACCTCACTGGTTACTGTCCCTAATTGGCCCTTCTTCAATCGCCATCCTCTCAAGTGGTTGGACCCAAGCGAAGCTGAGAAGCACCCTAAGC
probe_38	GCCACAGAAGGCTGTGGTTTTCAAGGACAAGAAGAAATCGTCCAAGAAGTAGGTTGGTTTTGTTAAATTTGACTGGTTTTTTAGTCTAAGCATTTGTTTGCTAGGGTTTTTAAATCTTCA
probe_39	CAATTTAATGGATGGAATGAAACGTTATGGGATGAATTGGGTGTTAGAATAACTTTGTTTTGCTATAAATTTTATACCTTTTAGACAATGCCATTCGAGTTTCAGTTAGTTTTTTTAACT
probe_40	GCATGTTTGAAGTGTTGGTTCTCGTTTATAGCTGTATTGATTTCTATTCATTGATGCTCGGACCATTGTTTCTGATTAGCTTTGCCCTCTCAATTGCTTTAAGTACTTCATTTTAAAAAA
probe_41	TTAAAAAACCAGTTATGGATGATCTTGATTTTAATTATGTTTCCTCCATTGATAGATTTTCTAAGCTTGTTTTGACATGTGCATCCTTTTTAATGTCATGCAGAATACAAGAGAGACAGC
probe_42	GAGACAGCGTTTGCTCTAAGGAAGTTGCCTCTGGTCAAGGCCAAGAGGTATTTGGAAGATGTTATGGCTCACAAACAGGCCATTCCATTCCGGCGTTTCTGTGGTGGAGTTGGGCGTACT
probe_43	GGCGTACTGCACAAGCAAAGAACAGACACTCTAACGGACAAGGAAGATGGCCTGCCAAGTCTGCCAAGTTCATCCTGGATTTACTCAAGAATGCCGAGAGCAATGCTGAGGTATATCTAT
probe_44	ATATCTATCCTCTTATTTGTCTTCACTTTCTCATCAACTTCTGCTTGCCATTTTACTGACATTACTTACCTTTCCAGGTCAAGGGTTTGGATGTGGATGCACTCTACATTTCTCACATCC
probe_45	TCACATCCAGGTGAATCAGGCACAGAAGCAGAGACGTCGTACTTACCGGGCGCATGGAAGAATTAATCGTAAGCTTGTTTTGATTAGATTGGTATTAGTTAATCTTACAATTACATTTCC
probe_46	ACATTTCCATTTGTATTCACAGGACTCTTTTATTTTCCTTTCCAGCTTACATGTCCAGCCCTTGCCACATTGAGTTGACTTTATCTGAAAAGGAAGAGCCAGTTAAGAAAGAGGTAATTC
probe_47	GGTAATTCATTTCTTTCTAATTGGGTAGATGGTTTAATTAATATACCAGTGTTTTTTGGACCTTTCTAATTCTGTGTACTTGTATTGCAGCCTGAGACCCAGCTAGCAACCAGCAAGTCA
probe_48	GCAAGTCAAAGAAGTCTCAGGTTTCCTCTTGAGTGGCGATCTTAGTTCAGTGTGGATCCCTTTTTGATTTCTGTGTTTTTTTCTTAAGAAGGATTTTGTTGCGTATCTTATAGAATGATC
probe_49	GAATGATCAGATTTTTAGATGTACTGGACATCTTGTATGTTGATTTCGAATTATGTTTAGTTCTTTGTTTTATGAGAAATGTCTTGGCGAGTTTATTTGAAGTCTATTTTCATCACAGTT
