# Supplementary material for: Multiplex Editing of the Nucleoredoxin1 Tandem Array in Poplar: From Small Indels to Translocations and Complex Inversions
Source: CRISPR J. 2023 Aug 14;6(4):339–49. doi: 10.1089/crispr.2022.0096 (PMC10460964; doi:10.1089/crispr.2022.0096)
Supplement: Supplemental data [file Suppl_FigureS1.pdf]

A. *P. trichocarpa*

	PotriNRX1.1	*PotriNRX1.2*	*PotriNRX1.3*	*PotriNRX1.4*	*PotriNRX1.5**	*PotriNRX1.6*	*PotriNRX1.7*	*PotriNRX1.8*
PotriNRX1.1								
PotriNRX1.2	99%							
PotriNRX1.3	96%	96%						
PotriNRX1.4	95%	95%	96%					
*PotriNRX1.5**	89%	89%	93%	90%				
PotriNRX1.6	95%	95%	96%	98%	90%			
PotriNRX1.7	95%	95%	95%	95%	88%	95%		
PotriNRX1.8	96%	95%	97%	99%	90%	98%	96%	

B. *P. tremula x alba*

	PtaNRX1.1	*PtaNRX1.2**	*PtaNRX1.3*	*PtaNRX1.4*	*PtaNRX1.5*	*PtaNRX1.6*	*PtaNRX1.7*
PtaNRX1.1							
*PtaNRX1.2**	56%						
PtaNRX1.3	95%	69%					
PtaNRX1.4	99%	69%	96%				
PtaNRX1.5	98%	49%	94%	98%			
PtaNRX1.6	94%	68%	95%	94%	93%		
PtaNRX1.7	93%	69%	96%	94%	92%	96%	

Figure S1. Coding sequence identity matrix among poplar *NRX1* TAGs. A, *PotriNRX1s* from *P. trichocarpa.* B, *PtaNRX1s* from *P. tremula* x *alba* INRA 717-1B4. Asterisks indicate sequence truncation or alignment gaps.
