# Supplementary material for: Multiplex Editing of the Nucleoredoxin1 Tandem Array in Poplar: From Small Indels to Translocations and Complex Inversions
Source: CRISPR J. 2023 Aug 14;6(4):339–49. doi: 10.1089/crispr.2022.0096 (PMC10460964; doi:10.1089/crispr.2022.0096)
Supplement: Supplemental data [file Suppl_FigureS2.pdf]

```
PtaNRX1.3       GCAATTGTTCTAGCCTTTTGAGTAG-TTCATCACATAGCTAGCTATGGCCACCGAAGACG
PtaNRX1.4       GCAATTGTTCTAGCCTTTTGAGTAG-TTCATCACAGAGCTAGCTATGGCCACCGAAGACG
PtaNRX1.1       GCAATTGTTCTAGCCTTTTGAGTAG-TTCATCACAGAGCTAGCTATGGCCACCGAAGACG
PtaNRX1.5       GCAATTGTTCTAGCCTTTTGAGTAGTTTCATCACATAGCTAGCTATGGCCACCGAAGACG
PtaNRX1.2       GCAATTGTTCTAGCCTTTTGAGTAG-TTCATCACATAGCTAGCTATGGCCACCGAAGACG
PtaNRX1.7       GCAATTGTTCTAGCCTTTTGAGTAG-TTCATCAC----TTAACAATGGCCAACGAAGACG
PtaNRX1.6       GCAATTGTTCTAGCCTTTTGAGTAG-TTCATCAC----TTAACAATGGCCAACGAAGACG
                ************************* ********     ** * ******* ********

PtaNRX1.3       TTTCTCTCGACCTTTCAACGCTTCTTTCTTCAGAAGAGAGGGACTTTCTCATCCGCAACA
PtaNRX1.4       TTTCTCTGGACCTTTCAACGCTTCTTTCTTCAGTAGAGAGGGACGTTCTCATCCGCAACA
PtaNRX1.1       TTTCTCTCGACCTTTCAACGCTTCTTTCTTCAGAAGAGAGGGACGTTCTCATCCGCAACA
PtaNRX1.5       TTTCTCTCGACCTTTCGACGCTTCTTTCTTCAGCAGAGAGGGACTTTCTCATCCGCAACA
PtaNRX1.2       TTTCTCTCGACCTTTCAACGCTTCTTTCTTCAGCAGAGAGGGACTTTCTCATCCGCAACA
PtaNRX1.7       TTTCGCACGACCTTTCATCGCTTCTTTCGTCGGAAGAGAGGGACTTTCTCATCCGCAACA
PtaNRX1.6       TTTCGCACGATCTTTCATCCCTTCTTTCGTCGGAAGAGAGGGACTTTCTCATCCGCAACA
                **** *  ** *****  * ******** ** * ********** ***************

PtaNRX1.3       ATGGTGATCAGGTACTGCTTCTCTGATCTTATTCAAGTCTTCTTCTTGTCTTATTATAAG
PtaNRX1.4       ATGGTGATCAGGTACTGCTTCTCTGATCTTATTCAAGTCTTCTTCTTGTCTTATTATAAG
PtaNRX1.1       ATGGTGATCAGGTACTGCTTCTCTGATCTTATTCAAGTCTTCTTCTTGTCTTATTATAAG
PtaNRX1.5       ATGGTGATCAGGTACTGCTTCTCTGATCTTATTCAAGTCTTCTTCTTGTCTTATTATAAG
PtaNRX1.2       ATGGTGATCAGGTACTGCTTCTCTGATCTTATTCAAGTCTTCTTCCTGTCTTATTATAAG
PtaNRX1.7       ATGGTGACCAGGTACTGCTTTT---AGCTTAT----GGATTTCT----------------
PtaNRX1.6       ATGGTGACCAGGTACTGCTTTT---AGCTTAT----GGATTTCTCTGGTCTTAT-----G
                ******* ************ *   * *****    *  **  *

PtaNRX1.3       TTGA-------TAATTTTTATA-TGAACTTGTAGGTTTTTCTCGTAATTAAGATGAATAT
PtaNRX1.4       TTGACGTCTTTTAATTTTTATA-TGAACTTGTAGGTTTTTCTCGTAATTAGGATGAATAT
PtaNRX1.1       TTGACGTCTTTTAATTTTTATA-TGAACTTGTAGGTTTTTCTCGTAATTAGGATGAATAT
PtaNRX1.5       TTGACGTCTTTTAATTATTATA-TGAACTTGTAGGTTTTTCTCGTAATTAAGATGAGTAT
PtaNRX1.2       TTGACGTCTTTTAATTTTTATA-TGAACTTGTAGGTTTTTCTCGTAATTAAGATGAATAT
PtaNRX1.7       ----------------------------------GTTTTTCTCGTAATTAAGATGGATAT
PtaNRX1.6       CGATCTACTTCTACTTGTCTTATTAAACTTGCAGGTTTTTCTCGTAATTAGGATGGATAT
                                                  **************** ****  ***

PtaNRX1.3       CATGTTTAAATTAGACTGCAAGCCTTGCTGAAGAACAAATTTATGACCAATTTCTGAAGC
PtaNRX1.4       CATGTTTAAATTAGACTGAAAGCCTTGCTGAAGAACAAATTTATGACCAATTTCTGAAGC
PtaNRX1.1       CATGTTTAAATTAGACTGAAAGCCTTGCTGAAGAACAAATTTATGACCAATTTCTGAAGC
PtaNRX1.5       CATGTTTAAATTAGACTGTTAGCCTTGCTTAAGAACAAATTTATGACCAATTTCTGAAGC
PtaNRX1.2       CATGTTTAAATTAGACTGGTAGCCTTGCTTAAGAACAAATTTATGACCAATTTCTGAAGC
PtaNRX1.7       CATGTTTAAATTAGACTGCGAACCTTGCTGAAGAACAAGTTTATGACCAATTTCTAGAGC
PtaNRX1.6       CATGTTTAAATTAGACTGCGAACCTTGCTCAAGAACAAGTTTATGACCAATTTCTAGAGC
                ******************  * ******* ******** ****************  ***

PtaNRX1.3       TTTTTAATGATTAAGAAAAAGCTCCTTTTTTTGTGTTCCTTTTCTCACTGGCAAGAGAAA
PtaNRX1.4       TTTTTTATGATTAAGAAAAAGCTCCTTTTTTTTTGTTGCTTCTCTCACTGGCAAGAGAAA
PtaNRX1.1       TTTTTTATGATTAAGAAAAAGCTCCTTTTTTTTTGTTGCTTCTCTCACTGGCAAGAGAAA
PtaNRX1.5       -TTTTTATGATTAAGAAAAAGCTCCCTTTTTTTTGTTGCTTCTCTCACTGGCAAGAGAAA
PtaNRX1.2       -TTTTTATGATCAAGAAAAAGCTCCCTTTTTTTTGTTGCTTCTCTCACTGGCAAGAGAAA
PtaNRX1.7       --TTTTATGATTAAGAAAA---TCCTCAATTTTTGTTTACCCTCTTAAT-----------
PtaNRX1.6       --TTTTATGATTCAGAAAA---GCCTCAATTTTTGTTTACCCTCTTGAT-----------
                  *** *****  ******    **    *** ****     ***   *

PtaNRX1.3       GAAACCATGAATAAGATTTTGATTCGTTAAGGGCTG--ATG-TTTTTTTCTGATAATGTT
PtaNRX1.4       GAAACCATGAATAAAATTGTGATTCGTTAAGGGCTG--ATG-TTTTTTTCTGATAATGTT
PtaNRX1.1       GAAACCATGAATAAAATTGTGATTCGTTAAGGGCTG--ATG---TTTTTCTGATAATGTT
PtaNRX1.5       GAAACCATGAATAAAATTTTGATTCGTTAAGGGCTG--ATGTTTTTTTTCTGATAATATT
PtaNRX1.2       GAAACCATGAATAAAATTTTGATTCGTTAAGGGCTG--ATG-TTTTTTTCTGATAATGTT
PtaNRX1.7       --------------------------TTAAGGGCTA--ATG--TTTTTTCTGATTATGCT
PtaNRX1.6       --------------------------TTATGGGCTATTTTTTTTTTTTTCTGATTATGCT
                                          *** *****    *    ********** **  *

PtaNRX1.3       CGAGATTAATTTTTAGGTTAAGGTCAGCAATTTGGTTGGGAAGATTGTGGGCTTCTATTT
PtaNRX1.4       CGAGATTAATTTTTAGGTTAAGGTCAGCAATTTGGTTGGGAAGATTGTGGGGTTCTACTT
PtaNRX1.1       CGACATTAATTTTTAGGTTAAGGTCAGCAATTTGGTTGGGAAGATTGTGGGGTTCTATTT
PtaNRX1.5       CGAGATTAATTTTTAGGTTAAGGTCAGCAATTTGGTTGGGAAGATTGTGGGGTTCTACTT
PtaNRX1.2       CGAGATTAATTTTTAGGTTAAGGTCAGCAATTTGGTTGGGAAGATTGTGGGGTTCTACTT
PtaNRX1.7       CCACGCTAATTTT-AGGTTAGGGTCAGCAATTTGGTTGGGAAGATTGTGGGATTCTATTT
PtaNRX1.6       CGACACTAATTTTAAGGTTACGGTCAGCAATTTGGTTGGGAAGATTGTGGGATTCTATTT
                * *   ******* ****** ****************************** ***** **
```

```
PtaNRX1.3      CTCTGGTTCGTGGTGCGGGCCGTGCCGTAATTTCACTCCATTGTTGGTAGAAGTCTATGA
PtaNRX1.4      CTCTGGTTCGTGGTGCGGCCCATGCCGTAATTTCACTCCTTTGTTGGTAGAAGTCTATGA
PtaNRX1.1      CTCTGGTTCGTGGTGCGGCCCATGCCGTAATTTCACTCCTTTGTTGGTAGAAGTCTATGA
PtaNRX1.5      CTCTGGTTCTTGGTGCGGCCCATGCCGTAATTTCACTCCTTTGTTTGTAGAAGTCTATGA
PtaNRX1.2      CTCTGGTTCGTGGTGCGGCCCATGCCGTAATTTCACTCCTTTGTTGGTAGAAGTCTATGA
PtaNRX1.7      CTCTGGTTATTGGTGCGGGCCGTGCCGTAATTTCACTCCGTTGTTGGTAGAAGTCTATGA
PtaNRX1.6      CTCTGGTTCGTGGTGCGGGCCGTGCCGCAATTTCACTCCGTTGTTGGTAGAAGTCTATGA
               ********  ******** ** ***** *********** ***** **************

PtaNRX1.3      ACAGCTATCATCCAAAGGGGACTTTGAGGTGGTCTTCATTTCTTCTGACGGAGACGATGA
PtaNRX1.4      ACAGCTATCATCCAAAGGCGACTTTGAGGTGGTGTTCATTTCTTCTGACAGAGATGATGA
PtaNRX1.1      ACAGCTATCATCCAAAGGCGACTTTGAGGTGGTGTTCATTTCTTCTGACAGAGATGATGA
PtaNRX1.5      ACAGCTATCATCCAAAGGGGACTTTGAGGTGGTCTTCATTTCTTCTGATAGAGATGATGA
PtaNRX1.2      ACAGCTATCATCCAAAGGGGACTTTGAGGTGGTCTTCATTTCTTCTGACAGAGATGATGA
PtaNRX1.7      ACAGCTATCATCCAAAGGGGACTTTGAGGTGGTCTTCATTTCTTCTGACGGACACGATGA
PtaNRX1.6      ACAGCTATCATCCAAAGGGGACTTTGAGGTGGTCTTCATTTCTTCTGACGGAGACGATGA
               ****************** ************** **************  ** * *****

PtaNRX1.3      ATCCTTCAACACATACTTCTCCGAAATGCCCTGGCTTGCTATTCCCTTCTCTGATACGGA
PtaNRX1.4      ATCCTTCAACACATATTTCTCCGAAATGCCCTGGCTTGCTATTCCCTTCTCTGATACGGA
PtaNRX1.1      ATCCTTCAACACATATTTCTCCGAAATGCCCTGGCTTGCTATTCCCTTCTCTGATACGGA
PtaNRX1.5      ATCCTTCAACACATACTTCTCCGAAATGCCCTGGCTTGCTATTCCCTTCTCTGATACGGA
PtaNRX1.2      ATCCTTCAACACATATTTCTCCGAAATGCCATGGCTTGCTATTCCCTTCTCTGATACGGA
PtaNRX1.7      ATTCTTCAACACATACTTCTCCGAAATGCCATGGCTTGCTATTCCCTTCTCTGATACGGA
PtaNRX1.6      ATCCTTCAACACATACTTCTCCGAAATGCCATGGCTTGCTATTCCCTTCTCTGATACGGA
               ** ************ ************** *****************************

PtaNRX1.3      GACCCGCAAACGTCTTAAGGAAGTGTTCAAAGTAAGAGGGATCCCTACTCTTGTCATTTT
PtaNRX1.4      GACCCGCAAACGTCTTAAGGAAGTGTTCAAAGTAAGAGGGGTCCCAAATCTTGTCATTTT
PtaNRX1.1      GACCCGCAAACGTCTTAAGGAAGTGTTCAAAGTAAGAGGGATCCCAAATCTTGTCATTTT
PtaNRX1.5      GACCCGCAAACGTCTTAAGGAAGTGTTCAAAGTAAGAGGGCTCCCTAATCTTGTCATTTT
PtaNRX1.2      GACCCGCAAACGTCTTAAGGAAGTATTCAAAGTAAGAGGGATC-----------------
PtaNRX1.7      GACCCGCCAACGTCTTAAGAAAGTGTTCGAAGTAAGAGGGATCCCTAATCTTGTCATTTT
PtaNRX1.6      GAGCCGCCAACGTCTTCAGGAATTGTTCAAAGTAAGAGGGATCCCTAAACTCGTCATTTT
               ** **** ******** ** ** * *** *********** **

PtaNRX1.3      TGATACGAATGGCAAGGTTTCATGCGATGATGGAGTCAGCACTGTCATGGAACATGGCGT
PtaNRX1.4      TGATGCGAATGGCAAGGTTTCCTGCGATGATGGAGTCAGCACTGTCAAGGAACATGGCGT
PtaNRX1.1      TGATGCGAATGGCAAGGTTTCCTGCGATGATGGAGTCAGCACTGTCAAGGAACATGGCGT
PtaNRX1.5      TGATGCGAATGGCAAGGTTTCCTGCGATGATGGAGTCAGCACTGTCAAGGAGCATGGCGT
PtaNRX1.2      ------------------------------------------------------------
PtaNRX1.7      TGATACGAATGGCAAGGTTTCATGCGATGATGGAGTCAGCACTGTCATGGAACATGGCGT
PtaNRX1.6      TGATGCTAATGGTAAGGTTTCCTGCGATAATGGAGTCAGACTTGTCAAGGAACATGGCGT

PtaNRX1.3      GGATGGGTATCCGTTCAACCTTGATAGACTGAATTTCCTGAAAGAGCAAGAAGAGAATGC
PtaNRX1.4      GGATGGGTATCCGTTCAACCTTGATAGACTGAATTTCCTGAAAGAGCAAGAAGAGAATGC
PtaNRX1.1      GGATGGGTATCCGTTCAACCTTGATAGACTGAATTTCCTGAAAGAGCAAGAAGAGAATGC
PtaNRX1.5      GGATGGGTATCCGTTCAACCTTGATAGACTGAATTTCCTGAAAGAGCAAGAAGAGAATGC
PtaNRX1.2      ------------------------------------------------------------
PtaNRX1.7      GGATGGGTATCCGTTCAACCTTGATAGACTGATTTTCCTGAAAGAGCAAGAAGAGAAAGC
PtaNRX1.6      GGATGGGTATCCGTTCAACCTTGATAGACTGAATTTCCTTAAAGAGCAAGAAGAGAATGC

PtaNRX1.3      TAAGAAGAATCAAACCATAAGCTCTATCTTGGTTTCCAGCTCACGTGATTATGTGATTTC
PtaNRX1.4      TAAGAAGAATCAAACCATAAGCTCTATCTTGGTTTCAAGCTCACGTGATTATGTGATTTC
PtaNRX1.1      TAAGAAGAATCAAACCATAAGCTCTATCTTGGTTTCAAGCTCACGTGATTATGTGATTTC
PtaNRX1.5      TAAGAAGAATCAAACCATAAGCTCTATCTTGGTTTCCAGCTCACGTGATTATGTGATTTC
PtaNRX1.2      ------------------------------------------------------------
PtaNRX1.7      TAAGAAGAATCAAACCATAAGCTCTATCTTGGTTTCCAGCTCACGTGATTATGTGATTTC
PtaNRX1.6      TAAGAAGAATCAAACCATAAGCTCTATCTTGGTTTCCAGCTCACGTGATTATGTGATTTC

PtaNRX1.3      AAATGATGGAAAAAAAGGTAGATGTTTTCATTCAATTAGCTAGTTTTGAAATTGAAATGAT
PtaNRX1.4      AAATGATGGAAAAAAAGGTAGATG-GTTCATTCAATTAGCTAGTTTTGAAATTGAATTGAT
PtaNRX1.1      AAATGATGGAAAAAAAGGTAGATG-GTTCATTCAATTAGCTAGTTTTGAAATTGAATTGAT
PtaNRX1.5      TAATGATGGAAAAAAAGGTAGATG-GTTCATTCAATTAGCTAGTTTTGAAATTGAATCGAT
PtaNRX1.2      ------------------------------------------------------------
PtaNRX1.7      AAATGATGGAAAAAAAGGTAGATG-GTT-ATTCAATTAGCTAGTTTTGAAATTGAATTGAT
PtaNRX1.6      AAATGATGGAAAAAAAGGTAGATG-TTTCATTCAATTAGCTAGTTTTGAAATTGAATTGAT
```

```
PtaNRX1.3      GATGCCATCATTCA--------CATTTTTGCTATCTGTAATGGCAGATCCCTGTGTTGGA
PtaNRX1.4      GATGCCATCATTCACTTTTTGCCTTTTTGGCTATCTGTAATGGCAGATCCCTGTGTTGGA
PtaNRX1.1      GATGCCATCATTCACTTTTTGCCTTTTTGGCTATCTGTAATGGCAGATCCCTGTGTTGGA
PtaNRX1.5      GATGCCATCATTCA--------CTTTCTTGCTATCTGTAATGGCAGATCCCTGTGTTGGA
PtaNRX1.2      ------------------------------------------------------------
PtaNRX1.7      GATGCCATAATTCA--------CATTTTTTCTATCTGTAATGGCAGATCCCTTTGTTGGA
PtaNRX1.6      GATGCCATAATTCA--------CATTTTTGCTATCTGTAATGTCAGATCCCTGTGTTGGA

PtaNRX1.3      GCTTGAAGGAAAATTGGTTGGCTTGTATTTTTCAATCCATAC------GATGTGCTGTGA
PtaNRX1.4      CCTTGAAGGAAAATTGGTTGGCTTGTATTTCTCAATCCATGCTCATAGGATGTGCCGTGA
PtaNRX1.1      CCTTGAAGGAAAATTGGTTGGCTTGTATTTCTCAATCCATGCTCACAGGATGTGCCGTGA
PtaNRX1.5      CCTTGAAGGAAAATTGGTTGGCTTGTATTTCTCAATCCATGCTCATAGGATGTGCCGTGA
PtaNRX1.2      ------------------------------------------------------------
PtaNRX1.7      CCTTGAAGGGAAATTGGTTGGCTTGTATTTTTCAATCCATAC------GATGTGCTGTGA
PtaNRX1.6      CCTTGAAGGAAAATTGGTTGGCTTGTATTTTTCAGCCCATGCTCATAGGATGTGCCGTGA

PtaNRX1.3      ATTCACTCCAAAACTAGTGGAATTGTATAAGACGCTCAAGGAAAAAAGAGAGAACTTTGA
PtaNRX1.4      ATTCACTCCTAAACTAGTTGAATTACACAAGCGGCTCAAGGAAAAAGGAGAGAACTTTGA
PtaNRX1.1      ATTCACTCCTAAACTAGTTGAATTACAAAAGCGGCTCAAGGAAAAAGGAGAGAACTTTGA
PtaNRX1.5      ATTCACTCCTAAACTAGTTGAATTATACAAGAGGCTCAAGGAAAAAGGAGAGAACTTTGA
PtaNRX1.2      ------------------------------------------------------------
PtaNRX1.7      ATTCACTCCAAAATTAGTGGAATTGTATAAGACGCTCAAGGAAAGAAGAGAGAACTTTGA
PtaNRX1.6      ATTCACTCCTAAACTAGTGGAATTGTATAAGACGCTCAAGGAAAGAAGAGAGAACTTTGA

PtaNRX1.3      AGTAGTCCTAATATCTCTAGACGACGAGGAAGAAGACTTCAAAGAGAGATTTGAGACAAT
PtaNRX1.4      AGTAGTCCTAATATCTCTAGACTTTGAGGAAAAACACTTCAAAGAGAGTTTTGAGACAAT
PtaNRX1.1      AGTAGTCCTAATATCTCTAGACTTTGAGGAAAAACACTTCAAAGAGAGTTTTGAGACAAT
PtaNRX1.5      AGTAGTCCTAATATCTCTAGACTTTGAGGAAAAACACTTCAAAGAGAGTTTTGAGACAAT
PtaNRX1.2      ------------------------------------------------------------
PtaNRX1.7      AGTAGTCCTAATATCTCTAGACGACGAAGAAGAAGACTTCAAAGAGAGTTTTGAGACAAT
PtaNRX1.6      AGTAGTCCTAATATCTCTAGACGACGAAGAAGAAGACTTCAAAGAGAGTTTTGAGACAAT

PtaNRX1.3      GCCTTGGTTGGCATTGCCATTTAAGGACAAGAGCTGCGAGAAGCTAGTGCGGTATTTTGA
PtaNRX1.4      GCCTTGGTTGGCATTGCCGTTTAAGGACAAGAGCTGCGAGAAGCTAGCGCGGTATTTTGA
PtaNRX1.1      GCCTTGGTTGGCATTGCCGTTTAAGGACAAGAGCTGCGAGAAGCTAGCGCGGTATTTTGA
PtaNRX1.5      GCCTTGGTTGGCATTGCCGTTTAAGGACAAGAGCTGCGAGAAGCTAGCGCGGTATTTTGA
PtaNRX1.2      ------------------------------------------------------------
PtaNRX1.7      GCCTTGGTTGGCATTGCCTTATAAGGACAAGAGCTGCGAGAAGCTAGTGCGGTATTTTGA
PtaNRX1.6      GCCTTGGTTGGCATTGCCGTTTAAGGACAAGAGCTGCGGGAAGCTAGTGCGGTATTTTGA

PtaNRX1.3      ACTTGGAACCATTCCTAATCTTGTCATAATTGGTCAAGATGGGAAGACTTTGAACCCAAA
PtaNRX1.4      ACTTAGAACCATTCCTAATCTTGTCATAATTGGCCAAGATGGGAAGACTTTGAACCCAAA
PtaNRX1.1      ACTTAGAACCATTCCTAATCTTGTCATAATTGGCCAAGATGGGAAGACTTTGAACCCAAA
PtaNRX1.5      ACTTAGAATCATTCCTAATCTTGTCATAATTGGCCAAGATGGGAAGACTTTGAACCCAAA
PtaNRX1.2      -------------CCTAATCTTGTCATAATTGGCCAAGATGGGAAGACTTTGAACCCAGA
PtaNRX1.7      ACTTAGAACCATTCCTAATCTTGTCATGATTGGCCAAGATGGGAAGACTTTGAACCCAAA
PtaNRX1.6      ACTTCGAACCATTCCTAAGCTTGTCATAATTGGCCAAGATGGGAAGACTTTGAACCCAAA
                            ***** ******** ***** ************************ *

PtaNRX1.3      TGTAGCTGAACTGATCGAAGAACATGGTATTGAAGCCTACCCATTTACACCGGAAAAGCT
PtaNRX1.4      TGTAGTTGAACTCATCGAAGACCATGGAATTGAAGCCTACCCATTTACACCAGAAAAGCT
PtaNRX1.1      TGTAGTTGAACTCATCGAAGACCATGGAATTGAAGCCTACCCATTTACACCGGAAAAGCT
PtaNRX1.5      TGTAGTTGAACTCATTGAAGACCATGGAATTGAAGCCTACCCATTTACACCGGAAAAGCT
PtaNRX1.2      TGTAGTTGAACTCATCGAAGACCATGGAATTGAAGCCTACCCATTTACACCGGAAAAGCT
PtaNRX1.7      TGTAGCTGAACTGATCGAAGAACATGGTATTGAAGCCTACCCATTTACACCGGAAAAGCT
PtaNRX1.6      TGTAGCTGAACTGATCGAAGAACATGGTATTGAAGCCTACCCATTTACACCGGAAAAGCT
               ***** ****** ** ***** ***** *********************** ********

PtaNRX1.3      TGACGAG-CTAGCTGCAATTGAAAAGGCAAAACTGGAATCGCAGACGCTTGAATCAGTTT
PtaNRX1.4      TGATGAG-CTAGCTGCAATTGAAAAGGCAAAACTGGAATCGCAGACGCTTGAATCAGTTT
PtaNRX1.1      TGAAGAG-CTAGCTGCAATTGAAAAGGCAAAACTGGAATCGCAGACGCTTGAGTCAGTTT
PtaNRX1.5      TGAAGAGCCTAGCTGCAATTGAAAAGGCAAAACTGGAATCGCAGACGCTTGAGTCAGTTT
PtaNRX1.2      TGATGAG-CTAGCTGCAATTGAAAAGGCAAAACTGGAATCGCAGACGCTTGAGTCAGTTT
PtaNRX1.7      TGACGAG-CTAGCTGCAATTGAAAAGGCAAAACTGGAATCGCAGACGCTTGAGTCAGTTT
PtaNRX1.6      TGACGAG-CTAGCTGCAATTGAAAAGGCAAAACTGGAATCGCAGACGCTTGAGTCAGTTT
               *** *** ******************************************** *******
```

```
PtaNRX1.3       TGGTAAATGGGGAAAATGATTTTGTGATTGACAAAAGTGGCTCCAAGGTAATT-TTCTTG
PtaNRX1.4       TGGTTAATGGGGAAAATGATTTTGTGATTGACAAAAGTGGCTCCAAGGTAATT-TTCTTG
PtaNRX1.1       TGGTTAATGGGGAAAATGATTTTGTGATTGACAAAAGTGGATCCAAGGTAATT-TTTTTG
PtaNRX1.5       TGGTTAATGGGGAAAATGATTTTGTGATTGACAAAAGTGGATCCAAGGAAATT-TTTTTG
PtaNRX1.2       TGGTTAATGGGGAAAATGATTTTGTGATTGACAAAAGTGGATCCAAGGTAATT-TTTTTG
PtaNRX1.7       TGGTTAATGGGGAAAATGATTTTGTGATTGACAAAAGTGGCTCCAAGGTAATTGTTTTTG
PtaNRX1.6       TGGTTAATGGGGAAAATGATTTTGTGATTGACAAAAGTGGCTCCAAGGTAATTGTTTTTG
                **** *********************************** ******* **** ** ***

PtaNRX1.3       TTTTATGCTAACCTCGCATGTTGTTATTGCATTACTTTCAATAACTTGCTTATTATCTCG
PtaNRX1.4       TTTTATGCTAACCTTGCATGTTGTGGTTGCATTACTTTCAATAACTTTCTTATCATCACG
PtaNRX1.1       TTCTCTGCTAACGTCGCATGTTGTTATTTCATTACTTACAATAACTTGCTTATTATCTCG
PtaNRX1.5       TTCTGTGCTAACATCGCATGTTGTTATTGCATTACTTTCAATAACTTGCTTATTATCTCG
PtaNRX1.2       TTCTGTGCTAACATCGCATGTTGTTATTGCATTACTTTCAATAACTTGCTTATTATCTCG
PtaNRX1.7       TTCTATGCTAACCTCGCATGTTGTTACCGCATTACTTTCAATAACTTTCTTATGATCACG
PtaNRX1.6       TTCTATGCTAACCTCGCATGTTGTTACCGCATTACTTTCAATAACTTTCTTATGATCACG
                ** * ******* * *********     ******** ********* ***** *** **

PtaNRX1.3       AATTGAATTCATTAGCAAAGG--------TATTTTTATCATTCAAATGGTTTACTC-TTG
PtaNRX1.4       AATTGAATTCATTAGCAAAGGG------TTTTTTTTATCATTTAAGTGGTTTGTTC-TTG
PtaNRX1.1       AATTGAATTCATTAGCAAAGG--------TATTTTTATCAGTCAAATGGTTTGCTC-TTG
PtaNRX1.5       AATTGAATTCATTAGCAAAGG--------TATTTTTATCATTCAAATGGTTTACTCTTTG
PtaNRX1.2       AATTGAATTCATTAGCAAAGG--------TATTTTTATCATTCAAATGGTTTACTCTTTG
PtaNRX1.7       AATTGAATTCATTAGCAAAGGGTTTTTTTTTTTTTTATCATTCAAATGGTGTGCTC-TTG
PtaNRX1.6       AATTGAATTCATTAGCAAAGGGTTTTTTTTTTTTTTATCATTCCAATGGTTTACTC-TTG
                *********************        * ********* *  * **** *  ** ***

PtaNRX1.3       ATTAGGTCCCAGTGTCTGATCTAGTTGGAAAGAACATTCTTCTTTACTTCTCAGCTCAAT
PtaNRX1.4       ATTAGGTCCGAGTGTCTGATCTAGTTGGAAAGAACATTCTTCTTTACTTCTCAGCTCAAT
PtaNRX1.1       ATTAGGTCCCAGTGTCTGATCTAGTTGGAAAGAACATTCTTCTTTACTTCTCAGCTCAAT
PtaNRX1.5       ATTAGGTCCCAGTGTCTGATCTAGTTGGAAAGAACATTCTTCTTTACTTCTCAGCTCAAT
PtaNRX1.2       ATTAGGTCCCAGTGTCTGATCTAGTTGGAAAGAACATTCTTCTTTACTTCTCAGCTCAAT
PtaNRX1.7       ATTAGGTCCCAGTGTCTGAACTAGTTGGAAAGAACATTCTTCTTTACTTCTCAGCTCAAT
PtaNRX1.6       ATTAGGTCCCAGTGTCTGAACTAGTTGGAAAGAACATTCTTCTTTACTTCTCAGCTCAAT
                ********* ********* ****************************************

PtaNRX1.3       GGTGCCCTCCTTGTCGTGCCTTTTTGCCCAAGCTAATTGAAGCATATCACACAATTAAAG
PtaNRX1.4       GGTGCCCTCCTTGTCGTGCCTTTTTGCCCAAGCTAATTGAAGCATATCACACAATTAAAG
PtaNRX1.1       GATGCCCTCCTTGTCGTGCCTTTTTGCCCAAGCTAATTGAAGCATATCACACAATTAAAG
PtaNRX1.5       GGTGCCCTCCTTGTCGTGCCTTTTTGCCCAAGCTAATTGAAGCATATCACACAATTAAAG
PtaNRX1.2       GGTGCCCTCCTTGTCGTGCCTTTTTGCCCAAGCTAATTGAAGCATACCACACAATTAAAA
PtaNRX1.7       GGTGCCCTCCATGTCGTGCCTTTTTACCCAAGCTAATTGAAGCATACCACACAATTAAAA
PtaNRX1.6       GGTGCCCTCCATGTCGTGCCTTTTTACCCAAGCTAATTGAAGCATACCACACAATTAAAA
                * ******** ************** ******************** ************ 

PtaNRX1.3       GAAAAGGCAATGCATTTGAGGTGATCTTCATCTCAAGTGACAGCGACCAATCCACCTTTG
PtaNRX1.4       GAAAAGGCAATGCATTTGAGGTGATCTTCATCTCAAGTGACAGCGATCAATCCACCTTTG
PtaNRX1.1       GAAAAGGCAATGCATTTGAGGTGATCTTCATCTCAAGTGACAGCGATCAATCCACCTTTG
PtaNRX1.5       GAAAAGGCAATGCATTTGAGGTGATCTTCATCTCAAGTGACAGCGATCAATCCACCTTTG
PtaNRX1.2       GAAAAGACAATGCATTTGAGGTGATCTTCATCTCAAGTGACAGAGATCAATCCACCTTTG
PtaNRX1.7       GAAAAGACAATGCATTTGAGGTGATCTTCATCTCAAGTGACAGAGATCAATCCACCTTTG
PtaNRX1.6       GAAAAGACAATGCATTTGAGGTGATCTTCATCTCAAGTGACAGAGATCAATCCACCTTTG
                ****** ************************************ ** *************

PtaNRX1.3       ACGAGTTCTATTCAGAAATGCCTTGGTTAGCCCTTCCATTTGGTGATGAAAGGAATCAAA
PtaNRX1.4       ACGAGTTCTATTCAGAAATGCCTTGGTTAGCCCTTCCATTTGGTGATGAAAGGAAACAAA
PtaNRX1.1       ACGAGTTCTATTCAGAAATGCCTTGGTTAGCCCTTCCATTTGGTGATGAAAGGAAACAAA
PtaNRX1.5       ACGAGTTCTATTCAGAAATGCCTTGGTTAGCCCTTCCATTTGGTGATGAAAGGAAACAAA
PtaNRX1.2       ACGAGTTCTATTCAGAAATGCCTTGGTTAGCCCTTCCATTTGGTGATGGAAGGAAACAAA
PtaNRX1.7       ACGAGTTCTATTCAGAAATGCCTTGGTTAGCCCTTCCATTTGGTGATGAAAGGAAACAAA
PtaNRX1.6       ACGAGTTCTATTCAGAAATGCCTTGGTTAGCCCTTCCATTTGGTGATGGAAGGAAACAAA
                ************************************************ ****** ****

PtaNRX1.3       TCCTGAGTCGGAAATTCAAAATTCAAGGCATTCCTGCAGCTGTATCGATTGGGCCAAGTG
PtaNRX1.4       TCCTGAGTCGGAAATTCAAAATTCAAGGCATTCCTGCAGCTGTATCGATTGGCCCAAGTG
PtaNRX1.1       TCCTGAGTCGGAAATTCAAAATTCAAGGCATTCTTGCAGCTGTAGCGATTGGCCCAAGTG
PtaNRX1.5       TCCTGAGTCGGAAATTCAAAATTCAAGGCATTCCTGCAGCTGTAGCGATTGGGCCAAGTG
PtaNRX1.2       TCCTGAGTCGGAAATTCAAAATTCAAGGCATTCCTGCAGCTGTAGCGATTGGCCCAAGTG
PtaNRX1.7       TCCTGAGTCGGAAATTCAAAATTCAAGGCATTCCTGCAGCTGTAGCGATTGGGCCAAGTG
PtaNRX1.6       TCCTGAGTCGGAAATTCAAAATTCAAGGCATTCCTGCAGCTGTAGCGATTGGGCCAAGTG
                ********************************* ********** ******* *******
```

```
PtaNRX1.3       GCCGGACCATTACGAAGGAAGCTCGGATGCACCTGACAGCTTACGGGGCAGATGCTTTTC
PtaNRX1.4       GCCGGACCATTACGAAGGAAGCTCGGATGCACCTGACAGCTTACGGGGCAGATGCTTTTC
PtaNRX1.1       GCCGGACCATTACGAAGGAAGCTCGGATGCACCTGACAGCTTACGGGGCAGATGCTTTTC
PtaNRX1.5       GCCGGACCATTACGAAGGAAGCTCGGATGCACCTGACAGCTTACGGGGCAGATGCTTTTC
PtaNRX1.2       GCCGGACCATTACCAAGGAAGCTCGGATGCACCTGACAGCTTACGGGGCAGATGCTTTTC
PtaNRX1.7       GCCGGACCATTACCAAGGAAGCTCGGATGCACCTGACAGCTTACGGGGCAGATGCTTTTC
PtaNRX1.6       GCCGGACCATTACCAAGGAAGCTCGGATGCACCTGACAGCTTAC-GGGCAGATGCTTTTC
                ************* ****************************** ***************

PtaNRX1.3       CATTTACCGAGGAGCATCTAAAGCAATTGGAGGAGGAGATTGAGGAAAAGGCAAAGGGGT
PtaNRX1.4       CATTTACCGAGGAACATCTAAAGCAATTGGAGGAGGAGATTGAGGAAAAGGCAAAGGGGT
PtaNRX1.1       CATTTACCGAGGAGCATCTAAAGCAATTGGAGGAGGAGATTGAGGAAAAGGCAAAGGGGT
PtaNRX1.5       CATTTACCGAGGAACATCTAAAGCAATTGGAGGAGGAGCTTGAGGAAAAGGCAAAGGGGT
PtaNRX1.2       CATTTACCGAGGAACATCTAAAGCAATTGGAGGAGGAGCTTGAGGAAAAGGCAAAGGGGT
PtaNRX1.7       CATTTACCGAGGAACATCTAAAGCAATTGGAGGAGGAGCTTGAGGAAAAGGCAAAGGGGT
PtaNRX1.6       CATTTACCGAGGAACATCTAAAGCAATTGGAGGAGGAGCTTGAGGAAAAGGCAAAGGGGT
                ************* ************************ *********************

PtaNRX1.3       GGCCAGAGAAAGTGAAACACGAACTT
PtaNRX1.4       GGCCAGAGAAAGTGAAACACGAACTT
PtaNRX1.1       GGCCAGAGAAAGTGAAACACGAACTT
PtaNRX1.5       GGCCAGAGAAAGTGAAACACGAACTT
PtaNRX1.2       GGCCAGAGAAAGTGAAACACGAACTT
PtaNRX1.7       GGCCAGAGAAAGTGAAACACGAACTT
PtaNRX1.6       GGCCAGAGAAAGTGAAACACGAACTT
                **************************
```

Figure S2. Multiple sequence alignments of *PtaNRX1* genes. Alignments were performed using MUSCLE (v3.8) provided by EMBL-EBI. Putative start and stop codons are shown in red and gRNA target sites in blue.
