# Supplementary material for: Multiplex Editing of the Nucleoredoxin1 Tandem Array in Poplar: From Small Indels to Translocations and Complex Inversions
Source: CRISPR J. 2023 Aug 14;6(4):339–49. doi: 10.1089/crispr.2022.0096 (PMC10460964; doi:10.1089/crispr.2022.0096)
Supplement: Supplemental data [file Suppl_TableS3.pdf]

Table S3. *PtaNRX1* gene models in the two 717 haplotype genomes

Short Name	Gene model	Genomic coordinates
**HAP1**		
PtaNRX1.1	PtXaTreH.10G046700	Chr10:7340821..7343607 reverse
PtaNRX1.2	PtXaTreH.10G046800	Chr10:7355740..7357791 reverse
PtaNRX1.3	PtXaTreH.10G046900	Chr10:7362547..7365400 reverse
PtaNRX1.4	PtXaTreH.10G047000	Chr10:7368504..7371759 reverse
PtaNRX1.5	PtXaTreH.10G047100	Chr10:7378710..7381267 reverse
PtaNRX1.6	PtXaTreH.10G047200	Chr10:7391387..7393805 reverse
PtaNRX1.7	PtXaTreH.10G047600	Chr10:7432694..7435394 reverse
$PtaNRX1p_{c10.1}$	not annotated	Chr10:7437455..7439765 reverse
$PtaNRX1p_{c8.2}$	not annotated	Chr08:13554619..13556719 forward
**HAP2**		
$PtaNRX1p_{c10.2}$	not annotated	Chr10:6698070..6699432 reverse
$PtaNRX1p_{c10.3}$	PtXaAlbH.10G043900	Chr10:6708418..6715147 reverse
$PtaNRX1p_{c8.1}$	PtXaAlbH.08G149800	Chr08:14214201..14214975 forward

Data were obtained from *Populus tremula* × *Populus alba* HAP1 v5.1 and *Populus tremula* × *Populus alba* HAP2 v5.1 genomes available at Phytozome v13 (https://phytozome-next.jgi.doe.gov/).
